# Profile Features of Emulsified Asphalt Mixture Containing Steel Slag Based on Laser Scanning

**DOI:** 10.3390/ma13122679

**Published:** 2020-06-12

**Authors:** Feng Wang, Peide Cui, Xiaoshan Zhang, Mujaheed Yunusa, Yue Xiao

**Affiliations:** State Key Laboratory of Silicate Materials for Architectures, Wuhan University of Technology, Wuhan 430070, China; wangfengfeng@whut.edu.cn (F.W.); Yunusamujaheed2015@gmail.com (M.Y.); Xiaoy@whut.edu.cn (Y.X.)

**Keywords:** profile indexes, emulsified asphalt mixture, laser texture scanner, skid resistance, aggregate morphology

## Abstract

Micro-surfacing (MS), made of emulsified asphalt, is the most commonly used preventive maintenance technology in asphalt pavement. However, the studies on profile features of MS based on aggregate morphology are few. This study evaluated the profile features of MS and its effect on skid resistance. The aggregate morphologies were first characterized and modified emulsified asphalt was prepared. The three-dimensional profile features of four kinds of MS samples were captured by laser texture scanner. Results illustrate that steel slag aggregate can be used to enhance the skid resistance of pavement surface and bring about larger profile indexes than basalt and limestone due to its angularity index and flatness values. Further aging of steel slag to eliminate free calcium oxide (f-CaO) is recommended before being used in pavement surface layer.

## 1. Introduction

Asphalt pavement is the preferred form of high-grade pavement surface structure with many advantages such as high driving comfort and easy construction, which is widely used in pavement engineering [1,2,3]. However, distresses including raveling, potholes, and cracking often occur on the asphalt pavements, making it need to be maintained [4,5]. Maintenance mileage accounted for more than 95% of Chinese highways at the end of 2019, manifesting numerous maintenance tasks. Therefore, the focus of pavement engineering will inevitably change from construction to maintenance of asphalt pavement, and maintenance has gradually become the research hotspot of road material [6].

Maintenance technologies are composed of corrective maintenance and preventive maintenance. Preventive maintenance is constructed before appearance of distress and is more cost-effective compared with corrective maintenance that is generally used after distress has developed [7,8,9,10]. It can effectively reduce degradation rate of service performance, improve skid resistance and reduce maintenance costs. Micro-surfacing (MS), made of emulsified asphalt and fine aggregate, is the most commonly used preventive maintenance technology in highways and airport runways, owing to its excellent skid resistance, short time of resuming traffic and function of repairing rutting [11,12,13]. Liu et al. [14] prepared MS samples with waterborne epoxy resin (WER) modified asphalt and the results indicated that MS prepared by WER can obviously improve rutting resistance. The effect of reclaimed asphalt pavement (RAP) on skid resistance of MS was investigated by Wang et al. [15]. It was found that there is an optimum RAP content where the skid resistance of MS can be effectively improved. Zalnezhad et al. [16] used steel slag to replace siliceous aggregate by 61% and MS mixture by 100%. The results manifested that steel slag has proper compatibility with emulsion and leads to a stronger rutting resistance. MS samples containing steel slag were prepared by Cui et al. [17] and it was found that addition of steel slag can obviously enhance skid-resistance performance of MS.

As a kind of surface layer that is contact with wheels, the skid resistance of MS directly determines the stability and safety of traffic [18,19,20]. Skid resistance stands for the friction force between tire and pavement surface during rotation. Aggregate morphology and gradation are two critical factors influencing skid resistance. However, the studies on the skid resistance of MS based on the aggregate morphology are few. Meanwhile, there are only two types of gradation, Type II and Type III, for MS, according to ISSA (International Slurry Surfacing Association), which are suitable for airport runways and filling wheel ruts, respectively. Just two kinds of gradations cannot meet the requirement of skid resistance under various service conditions, especially for roads with large traffic volume and load. On the other hand, although macro surface characteristics, such as indicators of skid resistance and mean texture depth (MTD) of MS, have been investigated, there is no research on the micro profile features of MS to our knowledge. The profile features determine the surface undulations and the location of peaks and valleys, so they cannot be ignored in involvement with skid resistance [21]. The objective of the research is to evaluate the profile features and skid resistance of emulsified asphalt mixture samples made of aggregates with various morphological characteristics. 

## 2. Materials

### 2.1. Aggregate and Asphalt

Three kinds of aggregates: limestone, basalt and steel slag, were used in this study. Limestone belongs to sedimentary rock and its main mineral component is calcite, which is a widely used construction material. Basalt is a kind of eruptive rock, with feldspar and pyroxene as the main mineral phases. It has low water absorption, good adhesion to asphalt and soundness, so it is usually applied in the surface layer of asphalt pavement. Steel slag is the by-product of steelmaking processes. As a typical solid waste, its main mineral composition is tricalcium silicate, in addition to dicalcium silicate, RO phase, dicalcium ferrite and free calcium oxide. Steel slag, instead of natural aggregate, in pavement engineering has become one of its dominating recycling methods in recent years [22,23,24]. The basic properties of three kind of aggregates are given in Table 1.

Emulsified asphalt does not need to be heated, and can be mixed with aggregates at room temperature, which is very convenient for construction. In this research, emulsified asphalt was prepared by colloid mill, which uses the high-speed relative motion between gears to fully emulsify and mix the asphalt, water and modifier (styrene–butadiene rubber, 3%). The basic properties of modified emulsified asphalt are exhibited in Table 2.

### 2.2. Slurry Mixture

**Figure 1:**
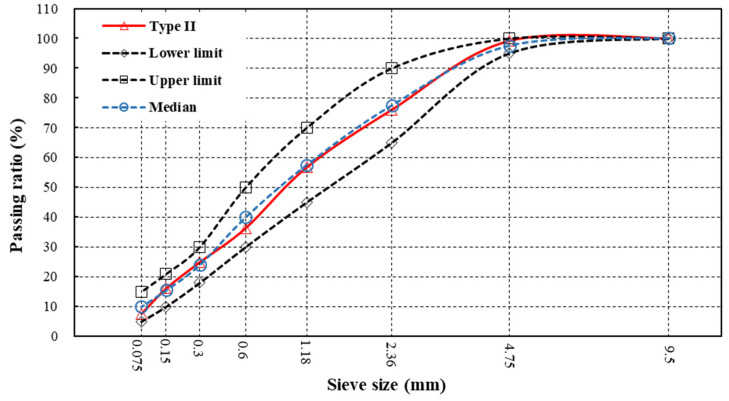
Gradation curve of Type II micro-surfacing samples.

The fundamental principle of gradation design is that coarser aggregates form a skeleton structure while fine aggregates are filled in the pores of the skeleton [25]. However, traditional gradations of MS, Type II and Type III (ASTM D 6372), cannot meet the requirement of skid resistance under various service conditions, especially for roads with large traffic volume and load. Therefore, uniformly-graded MS was designed and prepared in this study. There is only one size of aggregate in the sample, which makes no finer aggregate to fill in the air voids on the surface to reduce profile amplitude. However, the lack of fine aggregates packed in the framework has a negative impact on the mechanical stability of MS. Xiao et al. [21] found that the optimal size of aggregate for uniformly-graded MS is 1.18–2.36 mm. In consequence, basalt, limestone and steel slag with size of 1.18–2.36 mm were used to prepare uniformly-graded MS samples, respectively. In addition, MS made of basalt with Type II gradation was also designed to compare with uniformly-graded, as shown in Figure 1.

In addition to the aggregates and modified emulsified asphalt, water and cement were also added in the samples. Water can improve coating and mix workability, and curing of emulsified asphalt can be accelerated by cement, thereby reducing the time to resume traffic. Proportions of raw materials in 4 kinds of samples were determined according to the recommendation of ISSA 2017a guideline [26], and results are shown in Table 3. 

## 3. Research Methodologies

The following steps were conducted: (1) Modified emulsified asphalt was prepared and particle distribution of asphalt was evaluated by laser particle sizer (LPS). (2) The morphologies of basalt, limestone and steel slag were quantified by digital image processing. (3) Three-dimensional models and profile indexes of different uniformly-graded MS samples were evaluated and analyzed by laser texture scanner (LTS). (4) The relationship between profile features and skid resistance was established by linear-regression analysis. (5) Mechanical properties of MS samples were detected to assess the feasibility of MS with uniformly-graded.

### 3.1. Particle Distribution

The size and distribution of asphalt particles in emulsified asphalt have a considerable influence on its performance. The smaller the asphalt particles, the more dispersed they are in the emulsion and the better the storage stability. In this study, the LPS was used to investigate the size probability density and cumulative distribution of asphalt particles in the modified emulsified asphalt. The basic principle is that the laser is scattered by the asphalt particles when the laser passes through the emulsion. The detector will receive scattered lasers from different directions. Then, the optical and mathematical models are used to calculate the scattered values to obtain the particle distribution of asphalt.

### 3.2. Aggregate Morphology Features

Aggregate morphologies have significant influences on texture depth and skid resistance of asphalt pavement, which has been proved by previous studies [27,28,29]. Therefore, the morphological features of aggregates need to be adequately and accurately detected before preparing MS samples. There are many traditional methods to evaluate aggregate morphologies, such as ASTM D4791 (Test Method for Flat Particles, Elongated Particles, or Flat and Elongated Particles in Coarse Aggregate) and AASHTO T304 (Uncompacted Void Content of Fine Aggregate). Although they can be used to evaluate aggregate morphologies to some extent, they are affected by the subjective judgment of operators or are time-consuming. 

**Figure 2:**
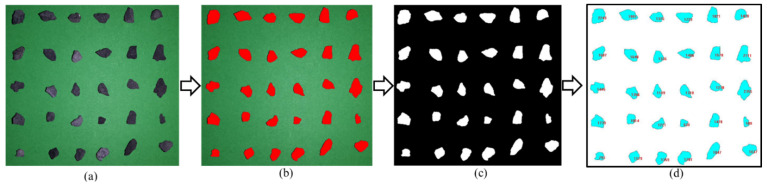
The recognition and analysis process of aggregates: (**a**) original image, (**b**) identified aggregates, (**c**) binarized aggregates, and (**d**) calculated aggregates.

**Figure 3:**
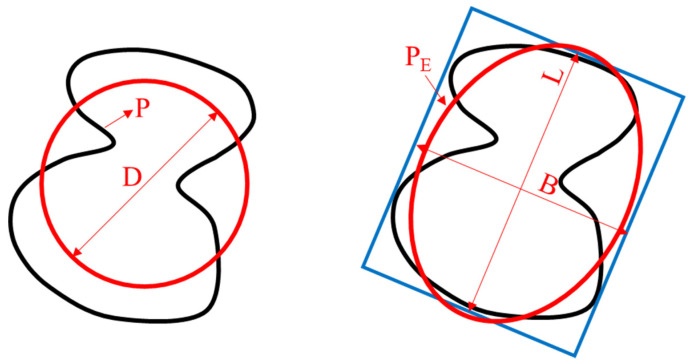
Illustration of aggregate feature parameters.

Digital picture processing technique was involved to detect aggregate morphologies rapidly and accurately in this study. The recognition and analysis are mainly divided into four steps. Firstly, aggregate is photographed by high resolution CCD camera. The color of image background should be obviously distinct from the aggregates so that they can be easily identified by image processing software, as shown in Figure 2a. Secondly, aggregates were recognized and selected, as the red area shown in Figure 2b. Then, Figure 2b was segmented and binarized into Figure 2c for easy access to aggregate outline and shape. Finally, aggregate feature parameters, including D, P, L, B and P_E_ were captured according to Figure 2d. The description and illustration of aggregate feature parameters are shown in Table 4 and Figure 3, respectively.

Three kinds of morphological characteristics, including flatness, angularity, and shape index were calculated based on aggregate feature parameters. Each morphological index was calculated by averaging 200 particles for each group of aggregates. Flatness reflects the ratio of particle dimensions as described in Equation (1). The greater the flatness, the flatter and more elongated the outline of the particles.

(1) $$\mathrm{Flatness}=\frac{L}{B}$$

Angularity indicates variations at the particle boundary and it was obtained by the ratio of perimeter of outline and equivalent ellipse according to Kuo et al. [30]. A higher value of angularity denotes a more angular outline.

(2) $$\mathrm{Angularity}=\frac{P}{P_{E}}$$

The shape of aggregate quantifies the relative form from 2D images of aggregate particles, as described in Equation (3). The perimeter of particle will be larger and shape index will be smaller if the overall shape of aggregate deviates from a circle, when the area is constant. Shape index is in the range of 0–1 and a perfect circle has a value of 1.

(3) $$\mathrm{Shape}\;\mathrm{index}=\frac{\pi D}{P}$$

### 3.3. Laser Scanning of Surface Profile

**Figure 4:**
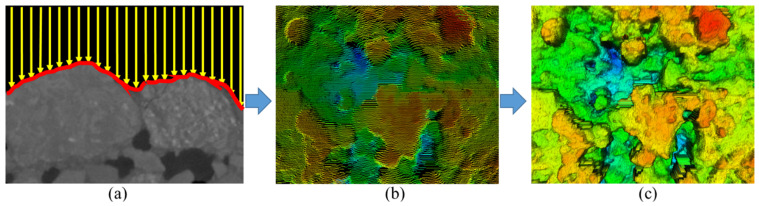
(**a**) One segment, (**b**) 1000 segments, and (**c**) 3D models.

The laser texture scanner (LTS 9400) was applied to detect profile features of MS samples, as shown in Figure 4a. Four profile indexes, including mean profile depth (MPD), height average (Ra), skewness (Rsk) and root mean square (RMS) were calculated on every segment, as presented in Figure 4b. Figure 4c shows the 3D surface model reconstructed by linking adjacent segments.

### 3.4. Skid Resistance and Mechanical Property

The skid resistance of MS samples were estimated by the British pendulum test in accordance with ASTM E303 [31]. The smaller the British Pendulum Number (BPN), the worse the skid resistance is. The wet track abrasion test (WTAT) was used to inspect the feasibility of uniformly-graded MS based on ISSA 100. Samples were abraded by a Hobart mixer for 300 s, and abrasion loss was calculated using Equation (4) according to ISSA A143.

(4) $${\mathrm{Abrasion}\;\mathrm{loss}=(m}_{1}{\;-\;m}_{2})/A$$

where, m_1_ = quality of specimen before abrasion (g); m_2_ = quality of specimen after abrasion (g); A = abrasion area (m^2^).

## 4. Results and Discussion

### 4.1. Particle Distribution of Emulsified Asphalt

**Figure 5:**
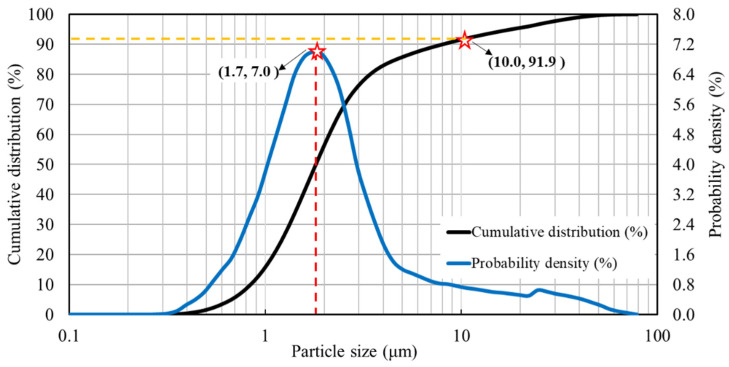
Cumulative distribution and probability density of emulsified asphalt.

Particle distribution of emulsified asphalt is presented in Figure 5. It was found that the particle size of asphalt in emulsion was concentrated in the range of 1–3 μm, and the size with the largest probability density was 1.7 μm, accounting for 7% of the total number of particles. Meanwhile, the particle size below 10 μm exceeds 90% of the total number of particles, indicating that the asphalt particles in the emulsified asphalt are well dispersed.

### 4.2. Aggregate Morphologies

**Figure 6:**
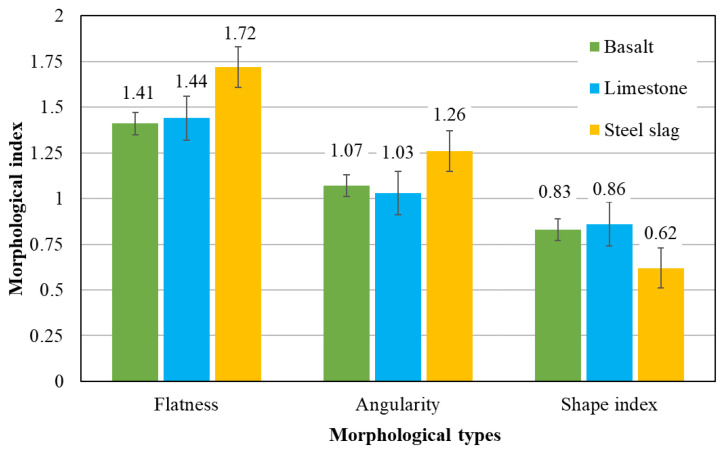
Aggregate morphologies of basalt, limestone and slag.

Flatness, angularity and shape index of limestone, steel slag and basalt are presented in Figure 6. The values of standard deviation are all less than 0.2, indicating a uniform division of morphological indexes. It was identified that flatness, angularity index and shape index of basalt are 1.41, 1.07 and 0.83, respectively, and those of limestone are 1.44, 1.03 and 0.86, which indicates that the morphologies of limestone and basalt are similar. The reason is because limestone and basalt, in this study, were conducted by the same crushing and shaping process. However, the soundness of limestone is smaller than that of basalt, so the effect of crushing has a certain impact on it, resulting in a slightly larger flatness and shape index.

In addition, the angularity index and flatness of slag are considerably larger, and the shape index is smaller than those of natural aggregates. This indicates that the morphology of slag is more abundant than the natural engineering aggregate, which signifies the edge sharpness is larger, and the shape is more non-circular. The reason is that the steel slag in this study were prepared by tank-type hot disintegrating method based on the thermal stress. High-temperature liquid steel slag undergoes rapid cooling after encountering water. The uneven cooling shrinkage stress causes the steel slag block to burst and decompose, resulting in steel slag having more abundant morphological characteristics than natural aggregates [32,33,34].

### 4.3. Three-Dimensional Surface Models and Profile Indexes of MS

**Figure 7:**
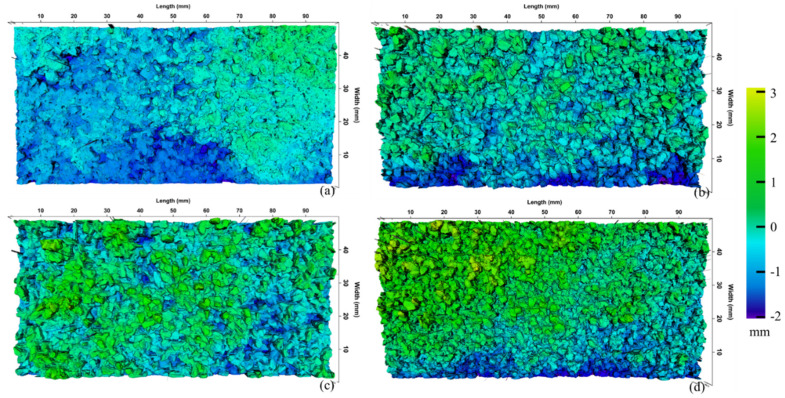
Colored 3D models of MS samples: (**a**) Type II, (**b**) MS-basalt, (**c**) MS-limestone, and (**d**) MS-steel slag.

There are totally 5600 scanning spots on each segment, and 1000 segments on the scanning area. Therefore, there are totally 5,600,000 scanning spots in the area of 100 mm × 50 mm. 3D models of MS’s surface were reconstructed by matching the spots’ heights to the different colors, as shown in Figure 7. It was found that that green, which represents a higher position, is the most widely distributed on samples made of steel slag and has the smallest distribution on Type II samples. It can be found from Figure 7 that uniformly-graded MS samples have an obvious graininess and higher texture depths compared with Type II. The reason is that fine aggregates were filled in the surface pores, which reduces the amplitude between peaks and troughs. Furthermore, surface texture features of three kinds of uniformly-graded samples were similar, whereas the general height of the MS-steel slag (d) is slightly higher than that of MS-basalt (b) and MS-limestone (c).

MPD, Ra, RMS, and Rsk were calculated to accurately analyze the surface profile features of MS. MPD and Ra are parameters directly describing the profile height, and RMS and Rsk describe the fluctuation of surface profile. The equations and results are shown in Table 5, where hi = ith height of spot on segment minus that of regression line, N = number of spots on each segment. 

MPD is a parameter for evaluating macro texture of pavement surface, and it is calculated on the basis of ASTM E1845. It can be identified from Table 5 that MPD of three types of uniformly-graded MS samples are all more than twice as large as that of Type II, implying that singleness size of aggregate can effectively increase texture depth. On the other hand, MPD values of uniformly-graded samples are close, 1.776, 1.794 and 1.863, for MS-basalt, MS-limestone and MS-steel slag, respectively. Moreover, since the edge slag is sharper and the shape is less circular than natural aggregate, steel slag can bring about a slightly larger texture depth on the surface than basalt and limestone.

Ra was found to have a similar rule with MPD. However, although the Ra values of three kinds of uniformly-graded samples are larger than that of Type II, they are less than twice as much as Type II, which means the increasing range of Ra is smaller than MPD. This is because the height of each scan spot on one segment was averaged in the calculation process of Ra, which reduces the effect of maximums and minimums in the result. Nevertheless, MPD is based on the peak on each segment, so the increase of texture depth is more prominent in the result of MPD.

**Figure 8:**
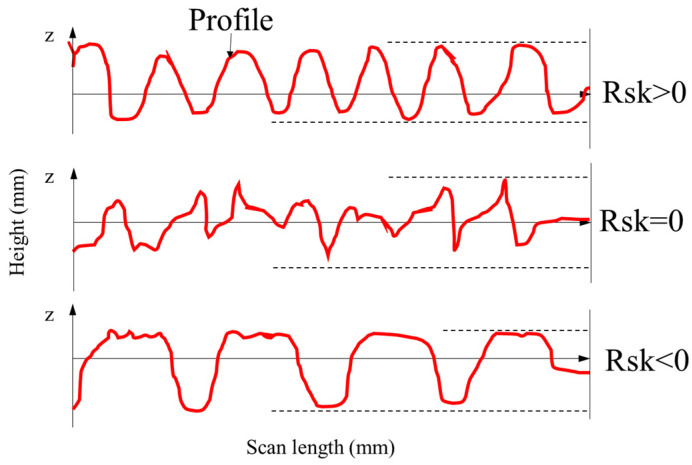
Schematic diagram of Rsk.

**Figure 9:**
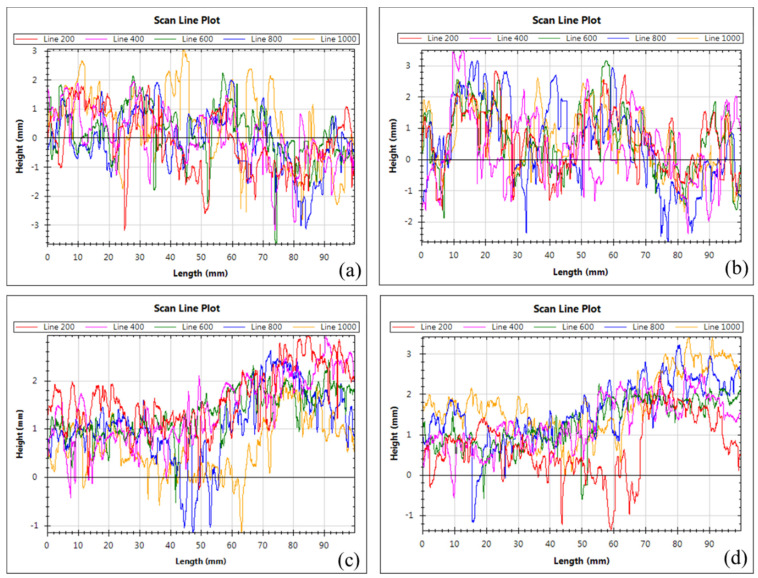
Scan line plots of (**a**) Type II, (**b**) MS-basalt, (**c**) MS-limestone, and (**d**) MS-steel slag.

RMS presents the diverge degree of surface profile from regression height. The trend of RMS is also similar to that of MPD. RMS values of MS-basalt, MS-limestone and MS-steel slag are 0.919, 0.997 and 1.013, respectively, while that of Type II is 0.505. Rsk stands for the symmetrical characteristic of surface profile. Positive numbers stand for the ratio of peaks is greater than valleys, while minus represents valleys dominate, as given in Figure 8. It can be found from Table 5 that the Rsk values of uniformly-graded samples are 0.332, 0.345 and 0.388 for MS-basalt, MS-limestone and MS-steel slag, respectively. However, Rsk value of Type II is −0.045. This manifests that singleness size of aggregate can significantly increase the positive texture on the surface of MS. Type II, MS-basalt, MS-limestone and MS-steel slag samples’ scan lines with number of 200, 400, 600, 800 and 1000 are shown in Figure 9a–d, respectively. It can be found that profiles of MS-basalt, MS-limestone and MS-steel slag are distributed above 0 overall, while those of Type II are mainly around 0, further confirming this conclusion.

### 4.4. Skid-Resistance and Mechanical Property

The BPN values of Type II, MS-basalt, MS-limestone and MS-steel slag are 54, 63, 59 and 64, respectively. This indicates that skid resistance of uniformly-graded samples is superior to Type II. In addition, the BPN value of MS-steel slag is the largest of four types of samples, in accordance with the results of profile indexes. 

**Figure 10:**
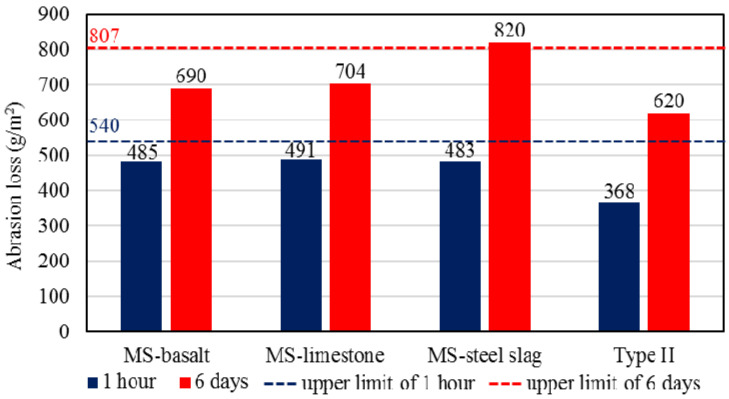
Abrasion loss of different kinds of samples.

The lack of fine aggregates packed in the skeleton structure has a negative effect on the mechanical stability of MS. Therefore, it is necessary to inspect the feasibility of aggregate with singleness size. The results of WTAT are shown in Figure 10. The abrasion loss of three kinds of uniformly-graded samples after 1 h are similar, with 485, 491 and 483 g/m^2^ for MS-basalt, MS-limestone and MS-steel slag, respectively. The 1-h result of Type II was significantly smaller than those of uniformly-graded, manifesting that singleness size of aggregate reduces the strength and wear resistance of the MS to some extent, whereas abrasion loss still meets the requirements of specification.

It can be seen from the 6-day results that the abrasion loss of Type II is also obviously less than those of uniformly-graded samples. However, the abrasion loss of MS-steel slag sample is greater than those of MS-limestone and MS-basalt, with a value of 820 g/m^2^, and exceeds the upper limit of the specification. The possible reason is that the free calcium oxide (f-CaO) in steel slag is converted into calcium hydroxide under the long-term contact with moisture, which causes the steel slag to expand in volume, thereby affecting the structural stability of MS-Steel slag. Therefore, steel slag needs to be aged to eliminate f-CaO before used as aggregate in MS more than hot-mix concrete. Outdoor aging is the widely used method to reduce f-CaO content, and slags in this study received 6 months aging treatment [35,36]. Furthermore, some studies altered mineral compositions of slag or added silica at high temperature to control f-CaO [37,38,39]. 

## 5. Conclusions

This study evaluated the profile features of MS samples and its effect on skid resistance. To this end, modified emulsified asphalt was prepared and morphologies of aggregates were detected. Three-dimensional models and profile features of four kinds of MS samples were captured by laser texture scanner (LTS). Relationship between profile indexes and skid resistance was established by linear-regression analysis. Finally, mechanical properties were detected to inspect the feasibility MS with uniformly-graded aggregates. Based on the results and analysis, the following conclusions can be drawn:

1. The color representing a higher position is the most widely distributed on 3D model of MS-steel slag and has the smallest distribution area in Type II samples. Uniformly-graded MS samples has an obvious graininess and higher texture depths compared with Type II.
2. MPD values of three types of uniformly-graded MS samples are all more than twice as large as that of Type II, implying that singleness size of aggregate can effectively increase texture depth. Ra and RMS were found to have the similar rule with MPD. Rsk values of uniformly-graded samples are positive, while that of Type II is −0.045, manifesting that singleness size of aggregate can significantly increase the positive texture on the surface of MS. Moreover, since the edge slag is sharper and the shape is less circular than natural aggregate, steel slag can bring about larger profile indexes than basalt and limestone.
3. Skid resistance of uniformly-graded samples is superior to Type II. The BPN value of MS-steel slag is the largest of four types of samples, which means the addition of steel slag can improve the skid resistance of MS samples.
4. The 1-h result of Type II was significantly smaller than those of uniformly-graded, whereas their abrasion loss still meets the requirements of specification. The abrasion loss of steel slag after 6 days is significantly larger than ordinary samples, and exceeds the upper limit of the specification resulting from the volume expansion of steel slag. Therefore, steel slag needs to be aged to eliminate f-CaO before used as the aggregate in MS.

## Figures and Tables

**Table 1:** Basic properties of three kinds of aggregate.

Properties	Basalt	Limestone	Steel Slag	Criterion
Sand equivalent (%)	68	71	75	ASTM D419
Soundness (%)	15.7	15.1	13.6	ASTM C88
Specific gravity	2.978	2.708	2.872	ASTM C127

**Table 2:** Basic properties of modified emulsified asphalt.

Properties	Performance	Criterion
Distillation (%)	61	ASTM D 7497
Softening point (°C)	56	ASTM D 36
Penetration (0.1 mm)	62	ASTM D 5
Ductility (cm)	>100	ASTM D 113
Charge	positive	ASTM D 244
Stability-24 h (%)	0.7	ASTM D 6930

**Table 3:** Material rates of four kinds of MS samples.

Slurry Mixtures	Abbreviation	Cement	Water	Asphalt Content
MS made of limestone	MS-limestone	1%	6.5%	5.1%
MS made of basalt	MS-basalt	1%	6.5%	4.8%
MS made of steel slag	MS-steel slag	1%	6.5%	5.5%
Type II	-	1%	7.5%	6.8%

**Table 4:** The aggregate features parameters and its description.

Parameters	Description
D	Diameter of the equivalent area circle
P	Length of aggregate’s outline
L	Length of major axis
B	Length of minor axis
P_E_	Perimeter of the equivalent ellipse

**Table 5:** Profile indicators of four kinds of MS.

Profile Index	Equation		Type II	MS-Basalt	MS-Limestone	MS-Steel Slag
MPD	$\frac{1}{2}[\max\left(h_{1}{,\ldots ,h}_{\frac{N}{2}}\right)+\max\left(h_{\frac{N}{2}+1}{,\ldots ,h}_{N}\right)]$	(5)	0.885	1.776	1.794	1.863
Ra	$\frac{1}{N}\sum_{i=1}^{N}{|h}_{i}|$	(6)	0.529	0.806	0.769	0.961
RMS	$\sqrt{\frac{1}{N}\sum_{i=1}^{N}h_{i}{}^{2}}$	(7)	0.505	0.919	0.997	1.013
Rsk	$\frac{1}{{\mathrm{RMS}}^{3}}\frac{1}{N}\sum_{i=1}^{N}h_{i}{}^{3}$	(8)	−0.045	0.332	0.345	0.388
